# Low preoperative hepcidin concentration is a risk factor for mortality but not for acute kidney injury after cardiac surgery

**DOI:** 10.1186/cc11009

**Published:** 2012-03-20

**Authors:** A Haase-Fieiltz, P Mertens, M Plaß, M Westerman, R Bellomo, M Haase

**Affiliations:** 1 Otto-von-Guericke University, Magdeburg, Germany; 2 German Heart Center, Berlin, Germany; 3 IntrinsicLifeSciences, La Jolla, CA, USA; 4 Austin Health, Melbourne, Australia

## Introduction

Hepcidin - expressed in renal proximal tubular cells - is a key regulator of iron homeostasis and was recently described as a renal biomarker that early postoperatively predicts protection from acute kidney injury (AKI).

## Methods

We studied 100 adult patients at increased risk of AKI (RIFLE) after cardiac surgery. Plasma and urine were sampled before induction of anesthesia and hepcidin 25-isoforms were quantified by competitive enzyme-linked immunoassay. Our objective was to assess the predictive indices of preoperatively measured urine and plasma hepcidin for the development of postoperative AKI and other patient-related outcomes, including the need for renal replacement therapy (RRT) and in-hospital mortality.

## Results

Preoperatively, patients not developing AKI presented with nonsignificantly higher urine and plasma hepcidin concentrations compared to patients that developed AKI which did not translate into a good predictive value for postoperative AKI (AUC-ROC <0.70 for both biomarkers). Also, the preoperative urine and plasma hepcidin concentrations as well as serum creatinine concentration did not distinguish patients requiring postoperative RRT from those who did not require RRT (urine: AUC-ROC 0.62 (95% CI 0.38 to 0.86), plasma: AUC-ROC 0.63 (95% CI 0.34 to 0.91), serum creatinine: AUC-ROC 0.61 (95% CI 0.22 to 0.99)). However, a low preoperative hepcidin concentration in urine (median 5 ng/ml, 25th to 75th percentiles 4 to 15 ng/ml) and in plasma (median 50 ng/ml, 25th to 75th percentiles 30 to 55 ng/ml) was a good predictor for postoperative mortality with an AUC-ROC for urine hepcidin of 0.89 (95% CI 0.73 to 0.99) (cut-off: 130 ng/ml, sensitivity 73% and specificity 100%) and an AUC-ROC for plasma hepcidin of 0.90 (95% CI 0.80 to 0.99) (cut-off: 55 ng/ml, sensitivity 83% and specificity 100%). Preoperative serum creatinine did not predict mortality (AUC-ROC 0.50 (95% CI 0.10 to 0.94). Patients who survived the hospital stay had a median preoperative hepcidin concentration in urine of 330 ng/ ml (25th to 75th percentiles 140 to 760 ng/ml), and plasma of 115 ng/ ml (25th to 75th percentiles 80 to 200 ng/ml).

## Conclusion

Our findings suggest that low preoperative hepcidin concentration indicates mortality but not renal endpoints in patients undergoing cardiac surgery. Thereby, hepcidin may contribute to early risk stratification. Findings should be validated in independent patient cohorts with a larger number of events.

